# Supplementary material for: Evaluating Effectiveness of YouTube Videos for Teaching Medical Students CPR: Solution to Optimizing Clinician Educator Workload during the COVID-19 Pandemic
Source: Int J Environ Res Public Health. 2021 Jul 2;18(13):7113. doi: 10.3390/ijerph18137113 (PMC8296861; doi:10.3390/ijerph18137113)
Supplement: Supplementary file 1 [file ijerph-18-07113-s001.zip › ijerph-1258722-supplementary.pdf]

Pre/Post test (English)

In adult basic life support, which of the following is the appropriate depth of chest compressions?
A) 1/3 of the depth of the anteroposterior chest diameter
B) Approximately 5 cm
C) Approximately 10 cm
D) 1/2 the depth of the anteroposterior chest diameter

In adult basic life support, which of the following is the appropriate rate of chest compressions?
A) 60 compressions per minute
B) 100 to 120 compressions per minute
C) 120 to 140 compressions per minute
D) 80 to 100 compressions per minute

What is the first thing you should do when you find an adult with suspected cardiac arrest?
A) Check the victim's consciousness.
B) Call for first aid.
C) Start chest compressions.
D) Ensure your own safety.

When the AED arrives at the victim with cardiopulmonary arrest, what is the first thing to do
A) Place the AED pad on the victim's chest.
B) Ensure the safety of the surrounding area.
C) Turn on the AED.
D) Check the casualty's pulse and breathing.

Which of the following correctly describes the method of chest compressions in adult basic life support?
A) Interruption of compressions is acceptable to ensure the rescue breaths.
B) The Interruption of chest compressions should be minimized.
C) Compressions should not be interrupted and should be continued even when tired.
D) May be performed with one hand if the rescuer has sufficient strength

Which of the following correctly describes the method of chest compressions in adult basic life support?
A) Depth is not necessary as long as the rhythm of the compressions is appropriate.
B) Rhythm is not important as long as depth is appropriate.
C) Allow complete recoil of chest wall after each compression.
D) Always apply compressions with your body weight.

Which of the following correctly describes the site of chest compressions in adult basic life support?
A) The upper half of the sternum or the middle of the line connecting the two axillae.
B) The left side of the chest.
C) The center of the chest or the lower half of the sternum.
D) Any site that does not compress the xiphoid process.

Which of the following is a correct description of agonal breathing?
A) It is not normal respiration, but respiratory function is preserved.
B) It is a sign of cardiac arrest and requires chest compressions.
C) The patient will improve if airway is secured.

D) If you are not sure whether the victim's breathing is agonal, consider that the patient is breathing spontaneously.

9. Which of the following should be done immediately after an AED electroshock?
A) Start chest compressions.
B) Check for consciousness.
C) Check the pulse.
D) Check for breathing

Which of the following is the appropriate sequential procedure of basic life support?
A) Check for consciousness, deliver a shock with an AED, and begin chest compressions.
B) Check the victim's consciousness, call for an ambulance and AED, check for breathing, and start chest compressions.
C) Check the victim's pulse, then check for consciousness, and deliver a shock with an AED.
D) Check the victim's consciousness, start chest compressions, deliver rescue breaths, and then check the pulse.

Pre/ Post test (Japanese)

1. 成人に対する一次救命処置において、胸骨圧迫の適切な深さは次のうちどれか。
A) 胸郭の 1/3 の深さ
B) 約 5cm
C) 約 10cm
D) 胸郭の 1/2 の深さ

2. 成人に対する一次救命処置において、胸骨圧迫の適切な速さは次のうちどれか。
A) 1 分間に 60 回
B) 1 分間に 100〜120 回
C) 1 分間に 120〜140 回
D) 1 分間に 80〜100 回

3. 心停止が疑われる傷病者を発見した際に、まず行うことは何か？
A) 傷病者の意識を確認する
B) 救急要請をする
C) 胸骨圧迫を開始する
D) 自分の安全を確保する

4. 心肺停止の傷病者にもとに AED が到着した際に、一番はじめにすることは何か？
A) AED パッドを傷病者の胸に貼る
B) 周囲の安全を確保する
C) AED の電源を入れる
D) 傷病者の脈拍と呼吸を確認する

5. 成人に対する一次救命処置での胸骨圧迫の方法について、正しく述べているものは次のうちどれか？
A) 確実に人工呼吸をするための圧迫中断は許容される

B) 胸骨圧迫の中断時間は最小限にする
C) 圧迫の中断は許されないため疲れた場合も継続する
D) 十分な力がある場合は片手で実施しても良い

6. 成人に対する一次救命処置での胸骨圧迫の方法について、正しく述べているものは次のうちどれか？
A) 圧迫のリズムが適切であれば深さは必要ない
B) 深さが適切であればリズムは重要ではない
C) 圧迫と圧迫の間は胸がもとに戻るようにする
D) 絶えず自分の体重をかけるように圧迫する

7. 成人に対する一次救命処置での胸骨圧迫の部位について、正しく述べているものは次のうちどれか？
A) 胸骨の上半分もしくは両腋窩を結んだ線の中央
B) 心臓の真上もしくは左胸部
C) 胸の中央もしくは胸骨の下半分
D) 剣状突起を圧迫しない部位であれば、どこの部位でも良い

8. 死戦期呼吸についての説明で正しいものは次のうちどれか。
A) 正常な呼吸ではないが呼吸機能は保持される
B) 心停止の兆候であり、胸骨圧迫の適応である
C) 気道確保をすれば改善する
D) 死戦期呼吸が否か判断できない場合は自発呼吸ありと考える

9. AED により電気ショックをした直後にすることは、次のうちどれか。
A) 胸骨圧迫を開始する
B) 意識を確認する
C) 脈拍を確認する
D) 呼吸を確認する

10. 心停止の傷病者に対する一次救命処置の適切な手順を次のうちどれか
A) 傷病者の意識を確認し、AED で電気ショックをして、胸骨圧迫を開始する
B) 傷病者の意識を確認し、救急車と AED を要請し、呼吸を確認し、胸骨圧迫を開始する
C) 傷病者の脈拍を確認し、次いで意識を確認し、AED で電気ショックを実施する
D) 傷病者の意識を確認し、胸骨圧迫を開始、人工呼吸を行った後に、脈拍を確認する
